# Mini-implant assisted rapid palatal expansion (MARPE) effects on adult obstructive sleep apnea (OSA) and quality of life: a multi-center prospective controlled trial

**DOI:** 10.1186/s40510-021-00397-x

**Published:** 2022-02-01

**Authors:** Daniel Paludo Brunetto, Christoph E. Moschik, Ramon Dominguez-Mompell, Eliza Jaria, Eduardo Franzotti Sant’Anna, Won Moon

**Affiliations:** 1 Federal University of Parana (UFPR) and private practice, Av Sete de Setembro 4456, Curitiba, Parana 80250-210 Brazil; 2 Private Practice, Munich, Germany; 3 Private Practice, Madrid, Spain; 4 The Forsyth Institute, 245 First Street, Cambridge, MA 02142 USA; 5 Department of Pedodontics and Orthodontics, Federal University of Rio de Janeiro (UFRJ), Rio de Janeiro, Brazil; 6 Department of Orthodontics, Institute of Oral Health Science, Ajou University School of Medicine, 206, World cup-ro, Yeongtong-gu, Suwon-si, Gyeonggi-do 16499 Republic of Korea

## Abstract

**Introduction:**

Transverse maxillary deficiency is a high prevalent growth disorder within the adult population that may lead to serious health issues, such as detrimental malocclusions and higher risk of developing obstructive sleep apnea (OSA). Mini-implant assisted rapid palatal expansion (MARPE), as it expands the mid-face and augment the nasal and oral cavities dimensions, may reduce the airflow resistance and thus play an important role on OSA therapy in some patients. The main objective of the present trial is to assess MARPE effects on the sleep and quality of life of non-obese adult OSA patients with transverse maxillary deficiency.

**Methods:**

A total of 32 participants were divided into intervention and control groups. They underwent physical evaluation, Epworth Sleepiness Scale (EES) and Quebec Sleep Questionnaire (QSQ), cone-beam computed tomography (CBCT) and home sleep testing (HST) for OSA before MARPE (T1) and 6 months after the intervention (T2).

**Results:**

Questionnaires EES (daytime sleepiness) and QSQ (OSA-related quality of life) presented significant statistical differences between the groups. We also found clinical and statistical (*p* < 0.01) differences between the groups regarding the apnea/hypopnea index (AHI), as well as others HST parameters (mean oxygen saturation and snoring duration).

**Conclusion:**

In our sample, MARPE (without any auxiliary osteotomy) showed a good success rate (85%) and promoted important occlusal and respiratory benefits. We observed important daytime sleepiness and OSA-related quality of life improvement, as well as the AHI (65.3%), oxygen saturation and snoring duration.

## Introduction

Obstructive sleep apnea (OSA) syndrome is one kind of sleep disorder, characterized by the partial or total obstruction of the upper airway and consequent airflow cessation during sleep, frequently leading to arousals and oxygen desaturations. A growing body of evidence has shown that these sleep disruptions and lower levels of blood oxygen may be responsible for an increased prevalence of the following conditions in OSA patients: arterial hypertension; cardiovascular morbidity and mortality; psychiatric disorders; type 2 diabetes; kidney malfunction; glaucoma and others [1, 2]. Patients also have higher risks of getting into car and work accidents because of their impaired concentrations as a result of inadequate sleep [3].

Alarming data has shown that the prevalence of OSA could climb up to 23.4% (95% CI 20.9–26.0) for female adults and 49.7% (95% CI 46.6–52.8) for male adults [4]. Recent studies showed that 80% of men and 93% of women are undiagnosed, and that these patients are twice as costly when compared to controls, mainly due to cardiovascular morbidity [4, 5]. Untreated OSA may have caused $3.4 billion additional medical cost in the U.S. in 1999 [6].

Due to its practicality and accuracy, sleep centers have recently increased their use of home sleep tests (HST). After reviewing validation studies, the American College of Physicians and the Canadian Sleep Society have encouraged HST for patients without medical comorbidities (e.g., pulmonary diseases, neurological disorders, and congestive heart failure) and concomitant sleep disorders (e.g., periodic limb movement and central apnea) [7]. Most HST's rely on type III monitors and include several important channels for an obstructive sleep apnea testing. These devices have been extensively tested and validated through in-lab polysomnography (PSG) comparison studies, exhibiting sufficient sensitivity and specificity [8–10].

Once diagnosed with OSA, patients should be immediately referred for treatment. Therapy modality usually depends on the severity of OSA, which is categorized accordingly to the number of respiratory events and clinical symptoms such as excessive daytime sleepiness, snoring, and witnessed apneas [11, 12]. Rapid palatal expansion (RPE), a well-documented auxiliary therapy for pediatric OSA, has been shown to broaden the nasal cavity and oropharyngeal dimensions after a mid-palatal suture split. When assessed by rhinomanometry and acoustic rhinometry, the increase in dimensions leads to a reduction in airflow resistance, presenting a significant improvement in functional breathing. Authors on a study on the topic demonstrated that RPE significantly reduced the apnea/hypopnea index (AHI) and the clinical symptoms in OSA children in the long term [13, 14]. More recently, a meta-analysis focusing on a total of 215 children with an average age of 6.7 years concluded that RPE appears to be an effective treatment for pediatric OSA [15].

Considering the adult population, surgically assisted rapid palatal expansion (SARPE) showed an increase in nasal cavity volume and functional improvement in breathing as well. Vinha et al. found an average 56.2% reduction in AHI and a significant improvement on daytime sleepiness in 16 patients who underwent SARPE [16]. Furthermore, recent studies demonstrated that a skeletal expansion could be achieved in determined young adults without the aid of osteotomies [17, 18]. This procedure is described as the Mini-implant Assisted Rapid Palatal Expansion (MARPE) [17]. Following the rationale regarding RPE and SARPE, we hypothesized that MARPE can lead to improvements in signs and symptoms of OSA. To our knowledge, there are no published trials assessing these effects.

The objective of our study is to assess the clinical outcomes of adult non-obese OSA patients who underwent MARPE, when compared to a control group, using home sleep testing and OSA-related quality of life questionnaires.

## Methods

The research project was submitted to and approved by the Federal University of Rio de Janeiro Institutional Review Board, under the protocol 80213017.4.1001.5257. Participants were recruited from the graduate orthodontic clinics of the following institutions: University of California – Los Angeles, USA; Federal University of Rio de Janeiro, Brazil; Federal University of Parana, Brazil; and Rey Juan Carlos University, Spain.

Inclusion criteria (both groups): presence of skeletal maxillary transverse deficiency (considering intermolar width measurements, posterior teeth buccolingual inclination, and posterior crossbites); presence of the upper first permanent molars; above 18 years old; voluntarily sign the written consent.

Exclusion criteria: presence of medical comorbidities (e.g., pulmonary disease, neurological disorders, and congestive heart failure); a body mass index higher than 35; presence of concurrent sleep disorders (e.g., periodic limb movement, central apnea, parasomnias, narcolepsy, insomnia, and circadian cycle disorders); systemic diseases; syndromes or severe craniofacial anomalies; pharyngeal pathology; previous orthodontic treatment; previous pharyngeal or nasal surgery (septoplasty, turbinectomy, and uvulopalatopharyngoplasty); concurrent treatment for sleep apnea.

### Study design

This is an intervention prospective controlled trial, with controls matched by AHI. Sample size calculations based on previous studies showed that eight subjects should be included into each arm of the trial, with a significance level of 0.05 and a study power of 80%, to detect a 9.5 variation on the apnea/hypopnea index [19, 20].

**Fig. 1:**
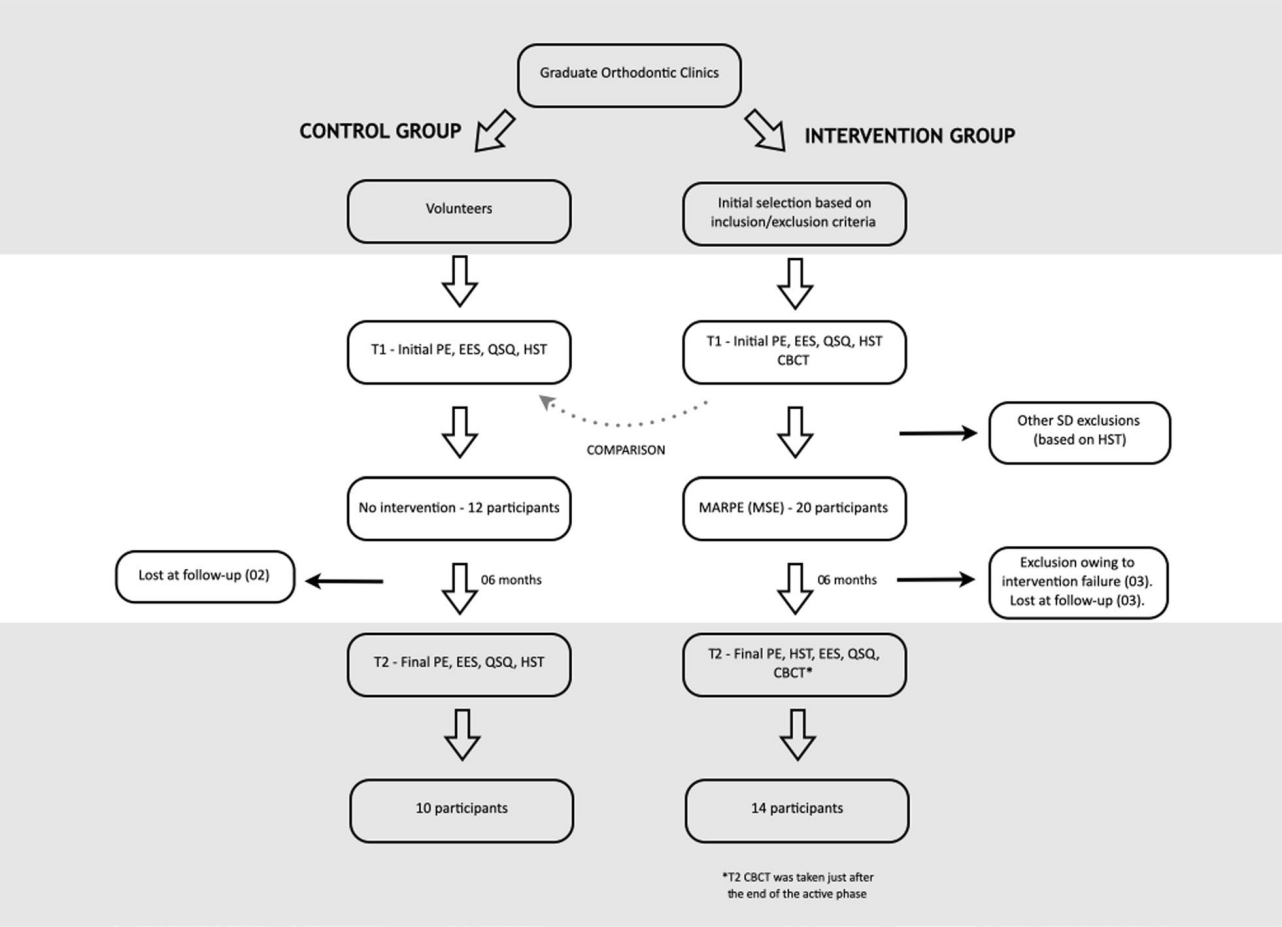
The study flow diagram. PE, Physical Evaluation; ESS, Epworth Sleepiness Scale; QSQ, Quebec Sleep Questionnaire; HST, Home Sleep Test; CBCT, Cone Beam Computerized Tomography; MSE, Maxillary Skeletal Expansion

If considered eligible regarding the inclusion/exclusion criteria, participants would undergo T1 exams in the following order: medical history and physical evaluation; Epworth Sleepiness Scale (ESS) and Quebec Sleep Questionnaire (QSQ); Home Sleep Test (HST); Cone Beam Computed Tomography (CBCT). Some sleep disorders may only be diagnosed by HST and, therefore, a few exclusion criteria were applied only after the first round of exams (Fig. 1). Participants underwent T2 exams only after the expander was removed and the desired transverse dimension was achieved, approximately six months after the last activation of the expander. The only exception was the CBCT, which was taken just after the last activation with the expander on the palate, in order to radiographically quantify the sutural split. Post-expansion CBCT was also used to verify if any undesired collateral bone fracture or nasal side effect occurred, considering patient safety.

Volunteers were recruited to participate as controls and were matched by OSA severity levels with the intervention group. These participants underwent the same set of exams as that of the intervention group, except for the CBCT. CBCT is a radiation-emitting exam, and as it had no benefits for the control-group participants, irradiating these participants would not be in compliance with the ALARA principle. Once their OSA diagnoses was confirmed by the HST, participants were referred to conventional treatment. The ones who refused conventional treatment, sufficiently informed of health consequences, had all the exams repeated six months later (except CBCT), matching the same conditions as the intervention group. Control participants did not receive any kind of treatment.

### Medical history and physical evaluation

An anamnesis screened for the following medical conditions: arterial hypertension, previous stroke, type 2 diabetes, or metabolic syndrome. Participants would not be accepted into this stage if they had previously reported pulmonary disease, neurological disorders, and congestive heart failure.

During physical evaluations, the following aspects were assessed: body mass index (BMI), neck circumference (NC), Mallampati pharyngeal classification, mandibular sagittal position, and overjet. All of the variables assessed in the study are summarized in Table 1.

**Table 1:** Exams and corresponding variables

Exams	Variables	Acronyms	Brief description
Physical evaluation	Body Mass Index	BMI	Weight/height^2^
	Neck circumference	NC	Largest diameter of the neck (mm)
	Mallampati classification	Mallampati	Classification of oropharynx soft tissue hypertrophy
	Mandibular Sagittal Position	MSP	Distance from Gnation to Nasion, 3 categories (retrusive, normotrusive, protrusive)
	Overjet	Overjet	Sagittal distance from the upper and lower incisors’ incisal borders (mm)
Questionnaires	Epworth Sleepiness Scale	ESS	Tool to assess daytime somnolence
	Quebec Sleep Questionnaire	QSQ	Tool to assess OSA-related quality of life (average of all domains)
		QSQ I	“Sleepiness” domain
		QSQ II	“Diurnal symptons” domain
		QSQ III	“Nocturnal symptons” domain
		QSQ IV	“Emotions” domain
		QSQ V	“Social interactions” domain
Home sleep test	Total sleep time	TST	Estimated total time of sleep
	Apnea/hypopnea index	AHI	Count of respiratory events per hour of sleep
	Improvement index	AHI < 50%	Number of participants with an AHI 50% reduction after the intervention
	Improvement index II	AHI < 5	Number of participants with a post-intervention AHI of 5 or less
	Flow limitation index	FLOW	Percentage of sleep time with an important flattening of the breathing waves
	Blood O_2_ saturation index	SpO_2_	Mean fraction of saturated hemoglobin and total hemoglobin (%)
		SpO_2_min	Minimal fraction of saturated hemoglobin and total hemoglobin (%)
	Snoring	SNRdb	Intensity of oropharyngeal reverberation (db)
		SNR%	Time spent snoring versus total sleep time (min)
	Body Position	BPsup	Time spent on supine position versus total sleep time (min)
	Bruxism index	BRUX	Bruxism episodes per hour of sleep (#/h)
		BRUXap	Bruxism episodes related to apneic events per hour of sleep (#/h)
Cone-beam tomography	Intersutural gap	IG	Average of 3 measurements along the palate on an axial slice

Neck circumference was measured using a non-stretchable plastic measure tape in the midway of the neck [21]. Mallampati classification was assessed with the participant standing upright with the tongue protruding without phonation [22]. Mandibular sagittal position was defined by 5 mm thresholds regarding Gnathion’s (most inferior contour of the chin) position in relation to Nasion (deepest point of the superior aspect of the nasal bone), as follows: retrusive mandible (category 1), gnathion positioned 5 mm posteriorly; normotrusive mandible (category 2), gnathion within 5 mm discrepancy; protrusive mandible (category 3), gnathion 5 mm or more anteriorly positioned [23]. An overjet greater than 3 mm, which is the anterior–posterior or horizontal distance between the upper and lower incisors incisal borders, was considered excessive [22].

### Questionnaires

The questionnaires had two distinct objectives. First, they were used as a screening tool to identify the high risk of OSA and to consequently refer the participants to an HST. Secondly, they measured the impact of the intervention on the sleep variables assessed.

The epworth sleepiness scale (ESS) was chosen to assess the changes on the level of daytime sleepiness in response to the intervention. ESS is a simple, reliable, and self-administered questionnaire based on eight questions, some known to be very soporific and other less, where the participant must rate, using a 4-point hierarchy scale, what the chances of dozing off are for each situation [24]. The EES score is the sum of the values entered for each question, ranging from 0 to 24, where higher scores indicate greater daytime somnolence. Participants filled out the form before appliance delivery and six months after the active part of the expansion (after appliance removal).

The Quebec Sleepiness Questionnaire is a comprehensive, self-administered, and reliable tool to assess the OSA-specific quality of life aspects, specifically developed for clinical trials. The questionnaire consists of 32 items divided into five categories, namely Sleepiness, Diurnal symptoms, Nocturnal symptoms, Emotions and Social interactions, and each item is scored on a 7-point Likert scale. In this tool, however, a score of 1 represents the greatest impact on quality of life. All the categories are summed up and averaged at the end, resulting in a final total score, which represents the physiological and social consequences of OSA. This questionnaire was administered before appliance delivery and six months after the active part of the expansion.

### Home sleep test

Participants received detailed instructions by trained professionals to correctly assemble the NOX T3® (Nox Medical, Reykjavic, Iceland) monitor at home. They were oriented to reproduce the same sleep conditions across all the recordings (e.g., bedtime, location, room temperature, etc.), and to postpone the exam if there were any medical conditions at the time (e.g., influenza, tonsillitis). NOX T3 is a type III portable monitor that was calibrated to record the following channels/variables: apnea/hypopnea index, considered the primary outcome, derived from the airflow measured by the nasal cannula (nasal-oro pressure transducer) and respiratory effort—measured by thoracic and abdominal plethysmography belts; O_2_ blood saturation level (SpO_2_) measured by bluetooth oximeter (Model 3150, Nonin Medical Inc., Plymouth, MN); Flow limitation (FLOW), a mathematical algorithm built-in Noxturnal® software (version 3.0, Nox Medical, Reykjavic, Iceland), which measures the flattening of breath waves (derived from the flattening index from the European Sleep Research Society); snoring intensity (SNRdb) and snoring duration (SNR%), measured by built-in acoustic microphone; sleep bruxism (BRUX) and sleep bruxism related to apneic event (BRUXap), measured by electromyography with an electrode placed at the masseter; concurrence of time in supine position (BPsup), measured by the accelerometer; estimated total sleep time (TST). Automatic scoring was performed by Noxturnal® software, based on the 2012 American Academy of Sleep Medicine scoring criteria [25]. As there is no sleep staging on HST, the AHI and bruxism index were calculated using bedtime minus the period that the participant was presumably awakened (sleep latency) read by the actigraphy and other sleep indicators (e.g., participant report), deriving the total sleep time [10]. A board-certified sleep professional, who was blinded to groups of participants, manually edited the scores for interpretation accuracy improvement [26].

### Cone beam computed tomography

Scans were taken with an i-Cat (*Imaging Sciences International, Hatfield, PA*), with the voxel size set at 0.4 mm, 120 kV, 5 mA, and total scan time of 20 s. Participants were oriented to keep their teeth in gentle contact, to breathe smoothly, and to not swallow during the acquisition. DICOM files were imported into Dolphin Imaging® software (version 11.5; Dolphin Imaging & Management Solutions, Chatsworth, CA), in which multiplanar slices were assessed to individualize the treatment plan for each participant.

CBCT objectives included determining the best MARPE position along the hard palate and the most appropriate dimensions for the mini-implants; determining MARPE success by visualizing radio translucency in the mid-palatal suture region; and quantifying the suture widening (intersutural gap, Table 1), which is an average of three measurements made along the hard palate in an axial slice on the palatal plane, previously and thoroughly described in Cantarella et al. [17]. Measurements were performed using Dolphin Imaging software.

### Intervention

**Fig. 2:**
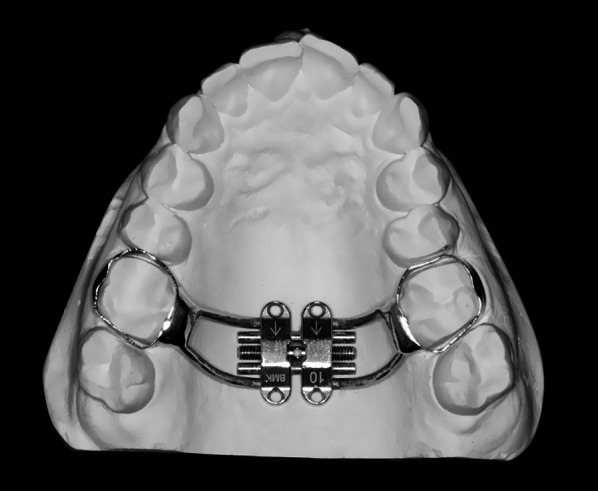
10 mm MSE ready for delivery

The Maxillary Skeletal Expander® (Biomaterials Korea, Seoul, South Korea) jackscrew was individually adapted for each participant in the laboratory, to assure its precise adaptation, using the following protocol [27]: fitting of orthodontic bands on permanent first upper molars; alginate impressions; bands transference into the impression and plaster pouring; wire bending and band soldering on the plaster casts (Fig. 2); expander intra-oral assembly; local infiltrative anesthesia; delivery of four 1.8 mm diameter and 9, 11 or 13 mm length orthodontic mini-implants (Biomaterials Korea, Seoul, South Korea), depending on the maxillary bone height.

**Fig. 3:**
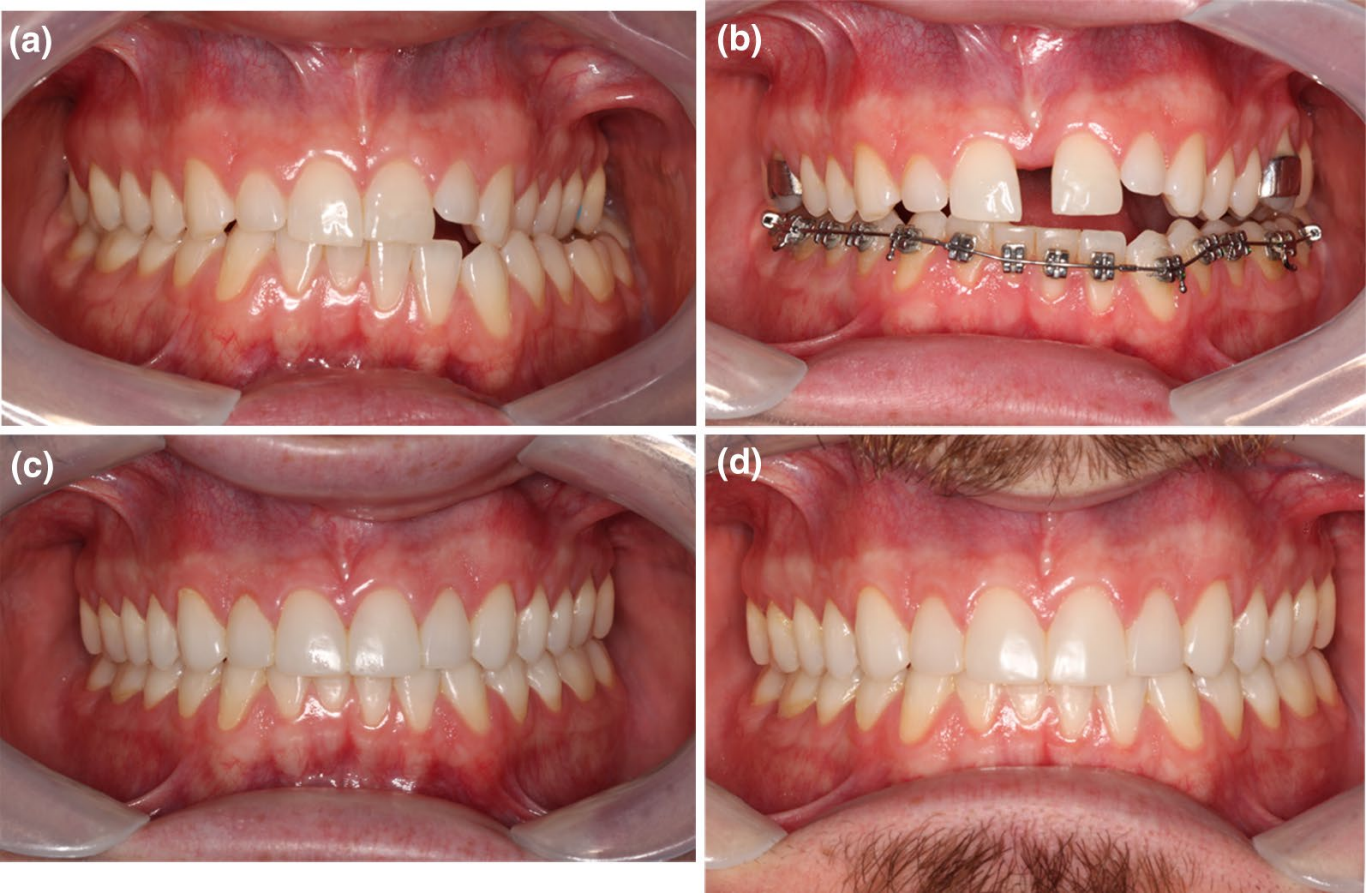
**A** 24-year old male patient with a maxillary transverse deficiency, a posterior cross-bite and OSA (AHI 19.7); **B** The malocclusion after 8.5 mm of palatal expansion (AHI 4.8); **C** just after the brackets debonding and restorative composites; **D** 2-years retention control

Participants were instructed to activate the expander at home, using the protocol: activate 0.5–1 mm per day until the interincisal diastema appears, and after that 0.25–0.5 mm per day. The jackscrew with the biggest expansion capacity (8, 10, or 12 mm) that had an excellent fit to the palate (flush to the mucosa) was individually chosen. If the participant had not reached enough palatal expansion at the first expansion, another MSE was delivered right after. Participants were seen every week to monitor progress and reinforce the hygiene/activation orientations. When the desired palatal expansion—defined as “the center of the upper alveolar ridge positioned 2–3 mm towards buccal compared to the center of the lower alveolar ridge”—was reached (Fig. 3), the expander was tied off and held in position for six months to allow for new bone formation. Total jackscrew opening was determined by the sum of the activations on the expander (0.25 mm each activation). No simultaneous orthodontic procedure was delivered at the retention period on the upper arch to avoid interferences with T1 exams. Intervention was considered successful if radio translucency was detectable at the post-expansion CBCT at the mid-palatal suture.

### Statistical analysis

As descriptive statistics, means and standard deviations were used for continuous variables and percentages for categorical variables. Wilcoxon signed-rank tests were used for intra-groups comparisons and the Mann–Whitney test was used for intergroup comparisons. SPSS software (version 17.0, Chicago, Ill) was used for the analyses, with statistical significance set at *p* < 0.05.

## Results

From February of 2016 to January of 2019, a total of 20 participants were recruited for the intervention group, three were lost at follow-up, and three were considered non-responsive to treatment. Hence, data from 14 participants were pooled for statistical analysis. The data of participants who had unsuccessful skeletal expansion (no sutural split and/or excessive tipping of posterior teeth) or were lost at follow-up (gave up treatment for personal reasons) were not considered. For the control group, 19 participants were tested and 12 presented an AHI higher than five. These 12 participants were recruited for the study and two were lost at follow-up, leaving ten participants for the analysis (Fig. 1).

Anthropometric data is displayed in Table 2. Only the overjet differed significantly between T0 and T1 in the intervention group. No significant weight or neck circumference variations were observed in both groups.

**Table 2:** Anthropometric data (means ± SD)

	Control		Intervention		Intergroups comparison T1 (Mann–Whitney)
	T1	T2	T1	T2	
Age	27.48 ± 4.01	–	28.36 ± 8.35	–	0.678
Gender (m/f)	6/4	–	6/8	–	–
BMI	28.05 ± 2.55	28.29 ± 2.44	28.59 ± 2.06	28.82 ± 2.14	0.098
NC	40.30 ± 2.54	40.11 ± 2.44	39.02 ± 3.17	39.26 ± 3.40	0.231
Mallampati	3.10 ± 0.73	–	3.20 ± 1.10	–	0.927
MSP	1.60 ± 0.51	–	1.42 ± 0.62	–	0.535
Overjet	4.04 ± 2.70	3.95 ± 2.57	3.23 ± 1.85	5.15 ± 1.95**	0.472

*Intra-group statistical significance (Wilcoxon signed rank) between T1 and T2, where: *p < 0.05; **p < 0.01; ***p < 0.001

Questionnaires’ results are summarized in Table 3. The control group did not exhibit significant differences between time points for either the ESS or all the QSQ categories, whereas the intervention group presented statistical differences for the ESS as well as almost all the QSQ domains (except social interactions).

**Table 3:** Questionnaires (means ± SD)

	Control		Intervention		T1 intergroups comparison (homogeneity)
	T1	T2	T1	T2	
ESS	11.73 ± 3.27	12.16 ± 3.90	12.87 ± 4.12	7.32 ± 3.61**	0.928
QSQ (total)	4.70 ± 0.95	4.92 ± 0.71	4.28 ± 1.10	5.52 ± 0.50*	0.120
QSQ I (sleepiness)	3.92 ± 1.06	4.12 ± 1.17	3.82 ± 1.26	5.46 ± 0.75*	0.529
QSQ II (diurnal symptons)	4.10 ± 0.99	4.25 ± 1.22	3.90 ± 1.12	5.26 ± 0.70*	0.629
QSQ III (nocturnal symptons)	5.29 ± 0.99	5.43 ± 0.81	4.36 ± 0.95	4.90 ± 1.12*	0.040*
QSQ IV (emotions)	5.17 ± 1.07	4.78 ± 1.26	4.35 ± 1.15	5.70 ± 0.60*	0.181
QSQ V (social interactions)	5.05 ± 1.23	5.34 ± 1.48	4.95 ± 0.80	5.14 ± 0.84	0.320

*Intra-group statistical significance (Wilcoxon signed rank) between T1 and T2 (displayed on T2 column), where: *p < 0.05; **p < 0.01; ***p < 0.001

**Fig. 4:**
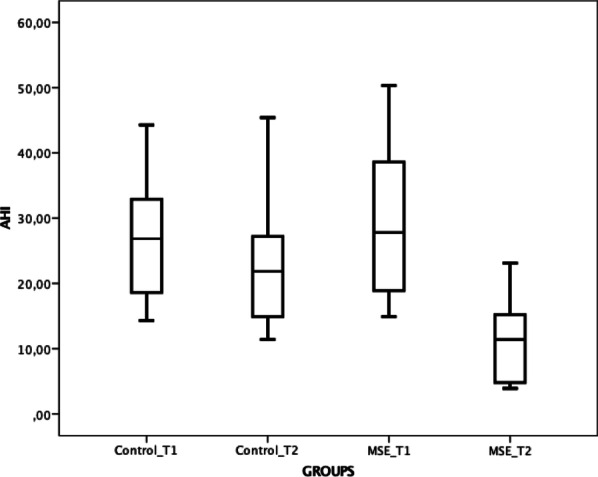
Boxplot comparing the AHI among the groups and timepoints

Statistics regarding the HST variables are summarized in Table 4 and Fig. 4. AHI was similar for both groups at baseline because they were matched using this variable. In contrast to the control group, however, the intervention group showed a statistical AHI reduction after the expansion. Of the participants who had successful expansion, 11 out of the 14 (78.5%) had a 50% reduction in the AHI. Five out of the 14 participants (35.7%) reached AHI < 5 events/hour. Four other participants also came close to that level, with their AHI around 8 events/hour. The participant with the most important reduction went from 32.4 to 7.7 (76.3%) events/hour. The participant with the least reduction went from 18.9 to 14.2 events/hour (24.9%). Oxygen saturation, snoring duration, and bruxism related to apnea also showed statistical improvements in the intervention group only.

**Table 4:** Home sleep test variables (means ± SD)

	Control		Intervention		T1 intergroups comparison (homogeneity)
	T1	T2	T1	T2	
Total Sleep Time	353.31 ± 53.63	356.02 ± 58.38	374.36 ± 68.14	368.02 ± 59.12	0.481
AHI	27.44 ± 10.35	24.18 ± 11.37	28.75 ± 11.39	11.45 ± 6.16**	0.798
AHI < 50%	–	0/10 (0%)	–	11/14 (78.5%)	–
AHI < 5	–	0/10 (0%)	–	5/14 (35.7%)	–
FLOW	10.80 ± 3.04	10.40 ± 2.91	11.33 ± 2.62	7.16 ± 1.68**	0.910
SpO_2_	91.23 ± 2.81	91.87 ± 2.25	91.92 ± 2.65	94.32 ± 1.97*	0.693
SpO_2_ min	85.50 ± 4.40	84.76 ± 5.37	84.10 ± 4.88	85.22 ± 3.82	0.859
SNR (db)	83.70 ± 3.71	83.30 ± 3.23	82.18 ± 5.16	81.36 ± 5.80	0.776
SNR (%)	13.10 ± 10.07	11.40 ± 8.20	15.68 ± 11.22	3.80 ± 3.43**	0.601
Body position	44.70 ± 12.71	41.80 ± 9.69	45.33 ± 14.20	48.89 ± 10.91	0.901
Bruxism	10.80 ± 3.04	7.61 ± 2.79	9.67 ± 3.72	7.02 ± 3.15	0.438
Bruxism/apnea	0.95 ± 0.42	0.97 ± 0.45	0.91 ± 0.50	0.40 ± 0.25*	0.540

*Intra-group statistical significance (Wilcoxon signed rank) between T1 and T2 (displayed on T2 column), where: * *p* < 0.05; ** *p* < 0.01; ***p < 0.001

The average suture opening (intersutural gap) for the intervention group was 6.61 ± 0.91 mm (95% CI 5.99–7.22), while the jackscrew average opening was 10.98 ± 2.53 mm (95% CI 9.65–12.29). There was no significant correlation between the amount of intersutural gap and the amount of AHI reduction (not dose-dependent). Thus, the null hypothesis—MARPE has no effect in the quality of life and in the objective sleep parameters of adult non-obese apneic patients—was rejected.

## Discussion

When considering the physical evaluation, we included some of the most important criteria routinely assessed in sleep studies. It is crucial to analyze whether there was a significant weight variation between the time points, because differences may point to confounding factors influencing the AHI [28]. For this reason, we used BMI and neck circumference—two simple and widely used indicators. A previous study reported that an OSA patient who was obese did not respond well to RPE, so we decided to limit the scope to non-obese adults [29]. Further, the mandibular sagittal position and overjet are described as risk factors for OSA. Hence, we used validated tools, such as cephalometry and pachymeter measurements, to assess these variables [23]. As expected, we found a significant overjet increase in the intervention group. This occurred due to the transient clockwise rotation of the mandible generated by premature occlusal contacts during buccal upper teeth expansion. Overjet will return to baseline values during the fixed appliance treatment.

The Epworth Sleepiness Scale was chosen for being one the most often utilized, simplest, and most validated tools to accurately assess daytime sleepiness [30]. It is also demonstrated to be an evaluative instrument, indicating that it has the power to detect somnolence variation between time points, making it especially useful in intervention clinical trials. To date, there are no trials similar to the one we presented here. Some related studies include Pirelli et al [14] which have found an important reduction in daytime sleepiness for pediatric patients subjected to rapid palatal expansion. Another related study, Vinha et al [16], found a reduction from 12.5 ± 5.3 to 7.2 ± 3.5 (*p* < 0.001) on the ESS scores of adults subjected to SARPE.

The Quebec Sleep Questionnaire, among many validated tools to assess OSA-related quality of life, was used because it is also an evaluative instrument [31]. Sleepiness, as a domain, had the greatest change, corroborating with the ESS results. “Social interactions” was the only domain that did not show significant improvement. This domain might have been influenced by the significant interincisal diastema that follows the expansion. Questionnaires were adopted in our study because it is also important to consider the patient’s perceived outcomes regarding the intervention, rather than simply restricting the analysis to objective measurements [32].

Several studies have directly compared HST monitors to PSG, and have found positive correlations between them. A recent study comparing NOX T3 and in-lab PSG found a sensitivity and specificity of 95% and 69%, respectively, when using the AHI ≥ 5 threshold [10]. For the AHI ≥ 15 threshold, sensitivity and specificity were 95% and 85%, respectively. The authors of the study concluded that despite some subtle differences, there was close agreement between the two exams. It is important to highlight that the HST may underestimate the AHI, because it is not able to detect arousals, which would be a component part of the index on an in-lab PSG [10].

An average reduction of 65.3% on the AHI was observed within the intervention group. Vinha et al. found a 56% AHI average reduction in SARPE patients, who underwent Le Fort I and mid-palatal osteotomies [16]. Recently, Liu et al. found an average 54% AHI reduction in 20 adults, non-obese patients subjected to maxillary distraction osteogenesis (DOME) with selective osteotomies, using a jackscrew associated with 4 to 6 mini-implants on the palate [33]. Similarly, Yoon et al. also found a significant reduction in the apnea index (17.65 + 19.30 to 8.17 + 8.47, *p* < 0.0001) and daytime somnolence (EES score 10.48 + 5.4 to 6.69 + 4.75), when analyzing 75 adults before and after a palatal expansion with a bone-borne expander [34]. We found a significant improvement on SpO_2_ levels, but not on the minimal SpO_2_. Vinha et al. reported a statistical difference in the SpO_2_ and oxygen desaturation index in SARPE patients [16, 33]. Cistulli et al. found a statically significant difference in minimal SpO_2_, from 89 ± 1 to 91 ± 1 (*p* < 0.05), but the authors studied a mixed sample of surgical and non-surgical patients [29]. None of our participants underwent any kind of osteotomy. Snoring duration presented an important improvement but not snoring intensity, showing a decrease in the pharynx airflow resistance. All participants were referred for further medical follow-up.

The current consensus is that sleep bruxism (SB) has a multifactorial etiology, with an array of biologic, psychologic, and exogenous causes [35]. Respiratory events and sleep arousals may be included as some of SB’s biologic factors, assuming that teeth grinding may be an attempt to maintain or restore the airway patency [36]. Tonic and phasic SB episodes are summed up and divided by total sleep time to arrive at the sleep bruxism index (events/hour). Sleep bruxism to apnea index is similar, but only the episodes following an apneic event are considered. We only found significant improvements only in episodes related to apneas, which is consistent with the AHI reduction in the intervention group. Bellerive et al [37], evaluating 36 children with in-lab PSG, found that most bruxers showed an average of 25% reduction in SB events after rapid palatal expansion. However, it is important to stress out that HST is not the gold standard for sleep bruxism assessment, because a previous individual bite force calibration is not performed, unlike PSGs.

MARPE in young adults can be considered a procedure with a high success rate, ranging from 87 to 100% depending on the technique [17, 18]. We found a MSE success rate of 85%. However, the evidence supporting non-surgical maxillary expansion in adults is still weak to moderate. The most frequent complication observed was mucosa inflammation around the mini-implants and the mini-implants lateral tipping, and five were lost due to mechanical instability. The mini-implant losses occurred in the retention period and did not interfere with expansion results. We did not observe any more serious complications, such as oro-nasal fistula, nasal mucosa inflammation, or bleeding in our participants. To date, there are no explanations or risk factors for the split failure observed in a few cases. One hypothesis may be that the low bone density that does not support the substantial mechanical forces applied to the mini-implants, leading to an increased inclination within the bone and molar buccal tipping. The average amount of jackscrew expansion was 10.98 ± 1.96 mm, keeping in mind that a couple of participants underwent two expansions. When a second expander was required to achieve the goals, the first jackscrew was removed and the second one was delivered at the following day, ensuring precise MARPE adaptations and no sutural ossifications. The mean gap observed at the suture, however, was 6.61 ± 0.91 mm, representing a 60% ratio in relation to total jackscrew opening, findings that are similar to those in Cantarella et al. (63%) [17].

There are many kinds of MARPE designs reported in the literature. They vary based on the type of the jackscrew, and the size and position of the mini-implants. When these variables change, all of the biomechanics within the expansion change as well. We chose the maxillary skeletal expander for this trial because of the evidence supporting the parallel expansion of the midpalatal suture when the jackscrew is positioned more posteriorly and the mini-implants have bicortical engagement [17, 38]. Theoretically, if there is a greater expansion in the posterior aspects of the maxilla, more effects are expected in the dimensions and the airflow resistance of the oro- and naso-pharynx.

Owing to OSA multifactorial etiology, patients may present varying degrees of responses to the expansion, as found in this study. We believe that patients with higher pharyngeal obstructions, such as in the nasopharynx, will be the ones yielding the best clinical outcomes. The maxillary expansion may influence the AHI by: reducing airflow resistance due to nasal cavity widening, allowing the tongue to reach for a more anterior position, due to the oral cavity expansion, and stretching of the soft palate muscles, thereby increasing their tonus and dynamics [16, 39]. In future studies, it would be of interest to detect the exact location of obstructions through a sleep endoscopy and confirm if this factor could influence the AHI response to the treatment. Even if MSE does not significantly decrease the AHI, reducing the airway resistance may facilitate the patient’s adherence to future CPAP therapy [40]. Recently, the American Association of Orthodontics released a white paper confirming the role of the orthodontist in the diagnosis and treatment of OSA [41]. The authors reported evidence supporting rapid palatal expansion as an alternative treatment to OSA in non-obese pediatric patients who were non-respondent to adenotonsillectomy. Using the same rationale, palatal expansion may become a relevant auxiliary therapy in OSA treatment of adult patients with transverse maxillary deficiency.

To our knowledge, this is the first trial to report that a specific type of MARPE (MSE), without any kind of osteotomies, can be used as an auxiliary in OSA treatments in non-obese adult patients with maxillary transverse deficiency. In addition to the respiratory benefits, the procedure is effective in correcting important occlusal aspects, such as posterior crossbites and arch perimeter deficiency [42].

Among the limitations of our study, the small sample size may be of concern. The participants were not randomized because we considered it unethical to not treat participants that knowingly needed expansions for occlusal purposes. The six-month follow-up can be considered a period with good stability, but these participants should ideally be seen for a few years after the intervention for long-term follow-ups. This is an ongoing trial and we will continue to follow the participants. HST has some disadvantages compared to in-lab PSG, such as the inability to assess sleep architecture and time spent on REM stage. On the other hand, its accessibility is better than that of a PSG, increasing the external validity of our study and reducing its replication costs.

## Conclusion

Based on the results obtained in the study, it is possible to conclude that:

- Maxillary skeletal expansion, MSE (a specific design of MARPE) is an efficient procedure for correcting maxillary transverse deficiency in non-obese young adults, yielding a success rate of 85%;
- participants who underwent MSE presented an important improvement in daytime sleepiness and OSA-related quality of life, as assessed by the validated questionnaires;
- the intervention group showed statistically significant improvements on the following sleep test parameters: apnea/hypopnea index (65.3%), mean oxygen saturation, snoring duration, and bruxism to apnea index. Approximately one-third (35.7%) of the intervention participants finished the trial with an AHI < 5;
- MSE, when appropriately indicated and conducted (associated or not with other therapies), may be considered as an auxiliary treatment for obstructive sleep apnea in non-obese young adults with a maxillary transverse deficiency.

## Data Availability

Data is available in machine-readable format, upon request.
